# Congenital Neonatal Intestinal Obstruction: Retrospective Analysis at Tertiary Care Hospital

**DOI:** 10.21699/jns.v5i4.393

**Published:** 2016-10-10

**Authors:** Vijay Singh, Manish Pathak

**Affiliations:** Department of Pediatric Surgery, RNT Medical College, Udaipur Rajasthan

**Keywords:** Neonate, Intestinal obstruction, Atresia, Malrotation

## Abstract

Background: The purpose of this study is to analyze the etiology, clinical presentation and outcome of neonatal intestinal obstruction at our institute.

Materials and Methods: The medical record of all the patients, presented with intestinal obstruction in neonatal period during 2014 and 2015 was reviewed retrospectively for etiology, clinical features, investigations, management, and outcome.

Results: Out of total 53 cases of neonatal intestinal obstruction, 27 were of intestinal atresia (9 cases (17%) were of duodenal atresia, 7 (13%) were of jejunal atresia and 8 (13%) were ileal atresias and 3 cases were found with colonic atresia); 7 were malrotation, 17 were Hirschsprung's disease (HD). All the patients were investigated with abdominal radiography and sonography. All patients were managed surgically. Overall mortality was 10/53 (18.8%). Out of 27 cases of atresia, 9 patients died (33% mortality). Septicemia was the cause of death in 7 patients (58.3%). Anastomotic leak was present in one mortality case.

Conclusion: The most common cause of neonatal intestinal obstruction is atresia. Duodenal atresia was the most common atresia in our study followed by ileal atresia. Postoperative complications like septicemia led to most of deaths in our series. Septicemia, wound infection, hypothermia, prematurity need special attention for survival of neonates.

## INTRODUCTION

Intestinal obstruction is the most common surgical emergency in the neonatal period. Early and accurate diagnosis of intestinal obstruction is paramount for proper patient management. It can be divided into– High intestinal obstruction like duodenal atresia, jejunal atresia, and malrotation or Low intestinal obstruction like colonic atresia, ileal atresia, Meckel's diverticulum with bands and HD. Vomiting is the chief complaint in high intestinal obstruction whereas abdominal distension with delayed or non-passage of meconium is the predominant complaint in low intestinal obstruction.[1-4] The purpose of this study is to analyze the etiology, clinical presentation, and outcome of neonatal intestinal obstruction at our institute.

## MATERIALS AND METHODS

It is a retrospective case series. The medical record of all the patients, presented with intestinal obstruction in neonatal period during 2014 and 2015 was reviewed retrospectively for etiology, clinical features, investigations, management, and outcome at RNT Medical College. Patients of anorectal malformation and esophageal atresia were excluded from the study. Cases of meconium ileus which were managed conservatively were also excluded.

Patients of duodenal atresia were treated with Kimura's duodeno-duodenostomy. Patients of jejuno-ileal atresia, colonic atresia and Meckel's diverticulum were treated by resection and anastomosis. HD was managed with initial colostomy; malrotation was managed by Ladd procedure.

## RESULTS

A total of 53 cases were treated from January 01, 2014 till December 31, 2015. Among them, 27 patients had intestinal atresia, 7 had malrotation, 17 had HD, and 2 had Meckel's diverticulum (Table 1). There were 40 males and 13 females (M: F- 3:1). Among males, nineteen had intestinal atresia, 14 had HD, 6 had malrotation, and 1 had Meckel's diverticulum. Age of presentation varied from 1 day to 24 days. Median age of presentation for patients with intestinal atresia was 3 days (age range 1-10 days), for HD was 7 days (age range 2-24 days), and for patients with malrotation it was 11 days (age range 2-19 days). Both patients with Meckel's diverticulum presented at day 2 of life. Median weight for patients with intestinal atresia was 2 Kg (1.2-3 kg), and for HD was 2.4 kg (1.6-2.8 kg). Median weight was 2.5 kg and 2 kg for malrotation and Meckel's diverticulum respectively. Forty-two patients (79%) had birth weight <2.5 kg and 15 patients were premature (28%). 

Twenty eight patients (53%) presented with bilious vomiting as chief complaint of these 24 patients had intestinal atresia. Rest of the 25 patients (47%) had abdominal distension and delayed passage of meconium as chief complaint; out of these 17 cases were found to have HD.

Intestinal atresia cohort comprised of 9 cases (17%) of duodenal atresia, 7 (13%) cases of jejunal atresia, 8 (13%) patients of ileal atresia, and 3 cases of colonic atresia. Type I was the most common type of duodenal atresia. In contrast, type III was the most common type in patients with jejunal, ileal and colonic atresia. Out of nine cases of duodenal atresia, 8 (89%) patients had type I while 1 (11%) had type III atresia. Out of 8 cases of ileal atresia, 1 (12.5%) had type I, 5 (62.5%) had type III, and 2 (25%) had type IV atresia. Out of 7 cases of jejunal atresia, 5 (71.5%) had type III and 2 (28.5%) had type IV atresia. All colonic atresia patients had type III atresia. Babies with atresia and Meckel's diverticulum presented earlier after birth while those with HD and other causes of obstruction presented late. Median age of presentation for intestinal atresia, HD, and malrotation was 3 days, 7 days and 11 days respectively. Median age of presentation for Meckel's diverticulum was 2 days. Most atresia patients (12/27- 44.4%) were premature as compared to only three premature patients in other group (3/26- 11.5%). 

Among these 27 cases of atresia, 8 cases (29.6%) had associated anomalies including anorectal malformation (ARM) in 2, volvulus in 3, tracheo-esophageal fistula in 1, malrotation in 1, and meconium ileus in 1. No associated anomalies were detected among patients having pathologies other than bowel atresia.

All patients were investigated with their respective modalities such as abdominal radiograph, ultrasound abdomen, upper and lower GIT contrast studies, laboratory investigations etc. All patients underwent their respective operations depending upon diagnoses. 

Sepsis (12 patients- 22.6%) and pneumonitis (4 patients- 7.5) were the postoperative non-surgical complications. Anastomotic leak (1 patient- 1.9%) and wound infection (1 patient- 1.9%) were surgical complications seen in this series. 

Overall postoperative mortality was 10/53 (18.8%); 9 patients died (33% mortality) were of intestinal atresia cohort; in rest of 26 cases, only 1 patient died (3.8% mortality). The mortality was attributed to only septicemia in 7 patients, pneumonitis in 2 patients, anastomotic leak in one patient and wound infection leading to septicemia in one patient. All the 10 deaths reported in this study also had birth weights <2.5 kg and 9 of these 10 mortality cases were premature (Table 1,2). Out of nine patients of intestinal atresia who died, 5 patients had associated anomalies (total 8 cases of intestinal atresia cohort had associated anomalies). Hypothermia, metabolic acidosis, and septic shock were main mortality events in our series.

**Figure:**
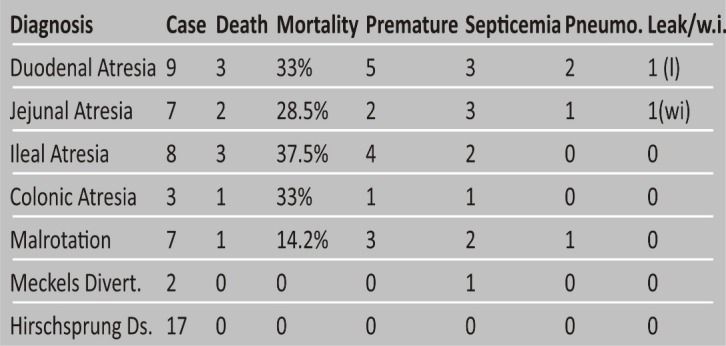
Table 1: Showing summary of study patients

**Figure:**
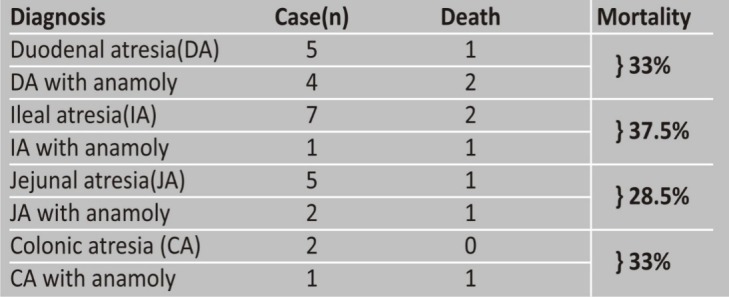
Table 2: Showing relationship of mortality with diagnoses.

## DISCUSSION

This series described our experience of managing neonatal intestinal obstruction over a period of two years. More than half of the patients in the study cohort had intestinal atresia as an etiology of congenital neonatal intestinal obstruction. Other etiologies were HD, Malrotation and Meckel's diverticulum with band obstruction. Duodenal atresia formed the most of cases in the patients with intestinal atresia. The incidence of duodenal atresia has been estimated as 1 in 6000-10,000 live births.[1] Boys are affected more commonly than girls. Eight of the nine patients with duodenal atresia were male in our series. More than 50% patients of duodenal atresia had associated congenital anomalies and classical double bubble sign on X-ray abdomen is enough to conclude the duodenal obstruction.[2] In our study 9 cases (33%) out of 27 were of duodenal atresia, occurring more commonly in boys and 5 cases had associated anomalies which is in agreement to the reported literature. 

Jejunoileal atresia occurs in approximately 1 in 5,000 live births, it occurs equally in males and females, and about one in three infants is premature.[3,4]. In our study, 7 cases had jejunal atresia and 8 cases had ileal atresia. Colonic atresia contributes 1.8-15% of all intestinal atresias, with an incidence of 1:40,000 live birth.[5]. In our study, three cases colonic atresia were encountered making an incidence of 11%. Contrast enema study is sometimes required to reach the diagnosis of colonic atresia. In most of the cases, primary resection and anastomosis is not possible due to the presence of distal short/micro colon and ruling out associated HD. If feasible, primary anastomosis is done otherwise staged procedure could be done. We had 3 cases of colonic atresia in which primary anastomosis was done; 1 case died out of 3. Hirschsprung's disease is present concomitantly in at least 2% of population. Some authors believe that it is imperative to rule out HD before reestablishing intestinal continuity in every patient with colonic atresia. We did not rule out HD in our patients with colonic atresia before primary anastomosis but the biopsy of the resected specimen and from the rectum showed presence of ganglion cells in all three cases.

HD is a developmental disorder characterized by absence of ganglia in the distal colon, resulting in a functional obstruction.[6] Its incidence is 1 in 5,000 live births. It is the second most common cause of intestinal obstruction after atresia in our study with a total of 17 cases out of 53. Intestinal obstruction due to midgut malrotation in newborns is well known entity. The incidence of malrotation is ∼1:500 births and the symptomatic incidence is 1:6000.[7,8] Any neonate presenting with sudden onset of bilious vomiting should be suspected for malrotation and volvulus. In our series, 7 cases had isolated malrotation while 1 case of sigmoid colonic atresia was associated with malrotation. All patients successfully underwent Ladd procedure as advocated by in 1932 by William Ladd.[9] Meckel's diverticulum is a rare cause of intestinal obstruction in neonates, 2 cases were reported in our study both were timely operated and survived.

Mortality is higher in atresia cohort in our series (33%). Prematurity, low birth weight, presence of other anomalies, postoperative septicemia, pneumonitis, anastomotic leakage and wound sepsis were considered various causes for mortality in our series.

## CONCLUSION

The most common cause of neonatal intestinal obstruction is atresia. Duodenal atresia was the most common atresia in our study followed by ileal atresia. Highest mortality was found in cases of atresia because most of these were premature and had low birth weight. Mortality is higher in patients of atresia with associated anomalies. Septicemia, wound infection, hypothermia, prematurity need special attention for survival of neonates.

## Footnotes

**Source of Support:** None

**Conflict of Interest:** None
